# Special Issue: Evolutionary Ecology of Venom

**DOI:** 10.3390/toxins13050310

**Published:** 2021-04-27

**Authors:** Kevin Arbuckle

**Affiliations:** Department of Biosciences, College of Science, Swansea University, Swansea SA2 8PP, UK; kevin.arbuckle@swansea.ac.uk

This Special Issue of *Toxins* aims to increase the profile and understanding of how ecology shapes the evolution of venom systems, and also how venom influences the ecological attributes of and interactions among species. The molecular biology, evolution, and pathology of venoms have long been fertile areas for research, particularly animal venoms of clinical significance. Some of this work has attempted to interpret biochemical and molecular evolutionary findings in the context of ecology, for instance, differences in venom composition being explained by some attributes that differ among species such as diet e.g., [1]. However, much rarer is an explicit attempt to study the evolutionary ecology of venom beyond simply using the ecological aspects as an ad hoc and post hoc explanatory framework.

Venom also presents a powerful system to address broader questions in evolutionary ecology thanks to its variability at multiple scales, its broad phylogenetic distribution, and its inherently tight link to key fitness components via natural enemy interactions [2]. Part of this utility as a model system is because venoms, by definition, operate at the molecule–ecology interface and so provide an unusually direct pathway linking genotype, phenotype, and the ecological theatre of evolution [3]. This combination of the high visibility of molecular variation to selection and involvement in antagonistic ecological scenarios has likely led to macroevolutionary effects on the lineages of venomous organisms, such as faster diversification rates [4].

Given that some now classic studies have linked venom to ecology (e.g., [5,6,7]) and it is a widely accepted phenomenon that selection pressures on the use of venom have influenced venom composition, why does the subject need the stimulus I hope this Special Issue will provide? The focus of much of the previous literature has concerned the relationships between snake venoms and diet (but there are exceptions, for instance [8]). While this is indeed an excellent example of venom evolutionary ecology, given the wide range of taxonomic diversity of venomous organisms and the ecological functions of venoms [2,9,10], there remains a substantial need to broaden out the scope and range of questions addressed. I hope that, at the very least, this Special Issue helps to illustrate that point.

Four papers deal with venom and diet, two focusing on spiders and two on snakes. Valenzuela-Rojas et al. [11] studied the venom of the wandering spider *Phoneutria boliviensis* in relation to sex and preferred prey classes, revealing that despite no difference in prey selection between males and females, males produced a smaller quantity of (equivalently potent) venom than females. Moreover, despite readily accepting both gecko and spider prey, the venom of *P. boliviensis* was far more lethal to geckos yet immobilized spiders quicker (at the same body size), demonstrating complex ecological relationships between diet and venom toxicity that may be difficult to infer from lethality data alone. Michálek et al. [12] similarly demonstrated the prey-specific toxicity of spider species, but also highlighted the effects of specialist diets. Specifically, they showed that the venom of specialist spiders was (unsurprisingly) more potent on their preferred prey. However, this appears to trade off with the ability to subdue alternative prey, since the venom of generalist spiders was more potent on other prey than that from closely related specialists. Such trade-offs suggest that interesting insights may arise from considering venom–diet relationships more broadly—rather than focusing largely on the prey-specificity of venom, relationships with dietary niche breadth may be important in venom evolution.

In the same vein, both papers considering snake venoms find evidence that diet breadth is an important but relatively poorly studied factor (certainly compared with prey-specific toxicity to particular prey species). Despite widespread support for prey-specific lethality and prey-specific toxins in the literature, Davies and Arbuckle [13] provide evidence that the *type* of toxicity (rather than its degree) has only limited association with the type of prey consumed, but rather that the diversity of toxicological effects is related to the diversity of prey in the diet. Lyons et al. [14] show that snakes with highly specialist diets drive the observed patterns of high prey-specific lethality, with dietary generalists showing a relatively constant degree of lethality with respect to different prey classes. The papers in this Special Issue therefore provide a new perspective on the links between diet and venom evolution by collectively suggesting an important but largely overlooked influence of diversity of diet on aspects of venom, rather than one-to-one relationships. Greater influence of diet breadth than specific prey items on venom composition has recently also been shown in populations of the cone snail *Conus miliaris* [8] and has interesting parallels with chemical defenses in musteloid mammals, which seem to be more strongly associated with ecological niche breadth than specific traits [15]. Overall, there is now growing evidence that niche breadth rather than specific attributes of the niche may be a key factor in the evolution of many traits involved in natural enemy interactions, including venom.

Parasitoid wasps use venom to incapacitate (but not kill) their host insects to allow their larvae to develop, and so the fitness of the wasps is directly dependent on the effective use of venom. Using an innovative experimental evolution approach, Cavigliasso et al. [16] investigated the evolution of venom composition in relation to host strains. Despite the hosts belonging to the same species, and thus only minor physiological variation may be expected, the venom composition of the parasitoid wasps evolved rapidly, showing host-specific changes in as little as four generations. This demonstrates the incredible potential of venom to evolve at high rates, which has already proven a challenge to estimation of ancestral states for animal venom data [17], suggesting it may be a general rule in venom evolution.

A large part of the wide interest in venom and venomous animals derives from the impacts of envenomation on humans, a defensive use of venom which has (for instance) contributed to the enormous global burden from venomous snakebite [18]. Yet despite this, very little emphasis has been placed on investigating whether antipredator defense has been an important driver of snake venom evolution. Using a large sample of envenomations by a wide range of snakes, and the assumption that defensive venoms should invoke early pain to deter predators before damage is inflicted, Ward-Smith et al. [19] tested the hypothesis that the defensive function has been important in venom evolution. They found no evidence that defense has been a pervasive influence in the evolution of venom in snakes overall, although interesting exceptions present fruitful avenues for further research [20].

More traditionally defensive venoms, those of hymenopteran insects, are widely known in terms of their effects on humans and the venom composition of several species. However, broader ecological associations with variation in venoms are still understudied. Yoon et al. [21] present data suggesting that factors such as sociality may have had an important impact on bee and wasp venoms: early indications well worth exploring more in future studies.

Venom is rare in mammals but presents an interesting study system in that intraspecific competition has been proposed as a primary use in several cases (otherwise rare in other venomous animal groups) [2,22]. In keeping with recent evidence that slow lorises use venom primarily in intraspecific competition [23], Nekaris et al. [24] present evidence that this has resulted in the evolution of contrasting face coloration that acts as an aposematic signal. Aposematic signals are usually directed at other species, so an example of warning signals directed primarily at conspecifics is unusual but may be linked to the common use of facial coloration as social signals in mammals, consistent with previous suggestions that sociality may be related to the evolution of mammal venoms [4]. This is an interesting parallel with the suggestion of sociality being important in the evolution of defensive venoms in hymenopterans mentioned earlier [21], and presents new research opportunities going forward.

Finally, this Special Issue presents two review papers which pull together many aspects of the evolutionary ecology of venoms and so provide broadly focused overviews of the subject area with a great many seed ideas for future research. Harris and Jenner [25] present a comprehensive overview of the venom systems of fishes. Unusually for reviews of animal venoms, there is only a brief outline of the biochemistry and morphology of venom systems (the focus of most reviews), and instead, it has a much stronger focus on the ecological drivers and evolutionary consequences of fish venoms. Schendel et al. [10] do not take a taxonomic focus but instead consider the diversity of animal venom systems across many levels of life (cf. [3]). This is a powerful perspective and enables the authors to highlight the functional, morphological, and behavioral diversity of venom systems (and the implications of such diversity), as well as give the first (to my knowledge) attempted estimation of the number of times venom has evolved across the animal kingdom.

I hope this Special Issue will enhance the profile of, and so stimulate further research towards an integrative understanding of, venom systems across levels of life. After all, venom is defined with emphasis on its ecological context, and a full understanding of venoms requires a multi-level program of research including both organismal and molecular toxinology.
